# Risk of Cancer after Lower Urinary Tract Infection: A Population-Based Cohort Study

**DOI:** 10.3390/ijerph16030390

**Published:** 2019-01-30

**Authors:** Chia-Hung Huang, Ying-Hsiang Chou, Han-Wei Yeh, Jing-Yang Huang, Shun-Fa Yang, Chao-Bin Yeh

**Affiliations:** 1 Institute of Medicine, Chung Shan Medical University, Taichung 402, Taiwan; hchiahung@gmail.com (C.-H.H.); hideka.chou@gmail.com (Y.-H.C.); 2 Division of Nephrology, Department of Internal Medicine, Lin Shin Hospital, Taichung 402, Taiwan; 3 Department of Medical Imaging and Radiological Sciences, Chung Shan Medical University, Taichung 402, Taiwan; 4 Department of Radiation Oncology, Chung Shan Medical University Hospital, Taichung 402, Taiwan; 5 School of Medicine, Chang Gung University, Taoyuan City 333, Taiwan; george66889@gmail.com; 6 Department of Medical Research, Chung Shan Medical University Hospital, Taichung 402, Taiwan; wchinyang@gmail.com; 7 Department of Emergency Medicine, School of Medicine, Chung Shan Medical University, Taichung 402, Taiwan; 8 Department of Emergency Medicine, Chung Shan Medical University Hospital, Taichung 402, Taiwan

**Keywords:** urinary tract infection, genitourinary organs cancer, retrospective cohort study

## Abstract

To investigate the association among lower urinary tract infection (UTI), the type and timing of antibiotic usage, and the subsequent risk of developing cancers, especially genitourinary cancers (GUC), in Taiwan. This retrospective population-based cohort study was conducted using 2009–2013 data from the Longitudinal Health Insurance Database. This study enrolled patients who were diagnosed with a UTI between 2010 and 2012. A 1:2 propensity score-matched control population without UTI served as the control group. Multivariate analysis with a multiple Cox regression model was applied to analyze the data. A total of 38,084 patients with UTI were included in the study group, and 76,168 participants without UTI were included in the control group. The result showed a higher hazard ratio of any cancer in both sexes with UTI (for males, adjusted hazard ratio (aHR) = 1.32; 95% confidence interval (CI) = 1.12–1.54; for females, aHR = 1.21; 95% CI = 1.08–1.35). Patients with UTI had a higher probability of developing new GUC than those without UTI. Moreover, the genital organs, kidney, and urinary bladder of men were significantly more affected than those of women with prior UTI. Furthermore, antibiotic treatment for more than 7 days associated the incidence of bladder cancer in men (7–13 days, aHR = 1.23, 95% CI = 0.50–3.02; >14 days, aHR = 2.73, CI = 1.32–5.64). In conclusion, UTI is significantly related to GUC and may serve as an early sign of GUC, especially in the male genital organs, prostate, kidney, and urinary bladder. During UTI treatment, physicians should cautiously prescribe antibiotics to patients.

## 1. Introduction

Urinary tract infection (UTI) is one of the most common infectious diseases and the main cause of community-acquired and nosocomial infections at admission [1]. Patients with UTI usually present with acute clinical UTI or chronic uncomplicated or complicated UTI. Complicated UTI involve individuals with a condition or more resistant pathogen that increases the risk of failing treatment with functional, metabolic, or structural abnormalities [2]. Moreover, the UTI incidence in women is much higher than that in men, but the majority are simple or uncomplicated UTI that occur in healthy or nonpregnant women [3]. In women, the urethra is close to the vagina and rectum, which can lead to the unintentional introduction of fecal flora into the urinary tract, the most common cause of frequent occurrence of UTI. Furthermore, the risk of bacteria colonization increases in postmenopausal women because of the loss of vaginal pH physiological tuning ability [4].

Lower UTI includes cystitis and prostatitis, and upper UTI include pyelonephritis. The Infectious Diseases Society of America observed a UTI prevalence of 1%–5% in healthy and premenopausal women and 1.9%–9.5% in pregnant women. Symptoms of lower urinary tract are common in men, and the prevalence increases with age. Up to 90% of men aged 50–80 years may suffer from troublesome lower urinary tract symptoms [5].

Inflammation is a main factor in cancer development, but studies on the relationship between UTI and cancer risks are scant [6]. A recent study demonstrated a relationship between UTI and genitourinary cancers (GUC) and confirmed that recurrent UTI are a risk factor for urinary bladder cancer [7]. Moreover, the relationship between UTI and other tumors is important because UTI-caused inflammatory response is a systemic symptom. In addition, UTI can be diagnosed on the basis of a combination of symptoms and a positive urine analysis or culture [8]. Thus, multiple antimicrobial therapies, including treatment with cephalosporin, quinolone, ampicillin, beta-lactam, amoxicillin, nitrofurantoin, sulfamethoxazole, and trimethoprim, can be prescribed [9,10]. Thus, using antibiotics is necessary for UTI treatment. Although evidence for the relationship between UTI and cancer risks is rare, it is reasonable to hypothesize that UTI disorders may still lead to cancers, especially GUC. Therefore, using a nationwide database, this study investigated the association between UTI and antibiotic usage influencing the risk of cancer.

## 2. Materials and Methods

### 2.1. Data Source

A national large-scale database, the Longitudinal Health Insurance Database (LHID), was used in this study. The 2010 LHID is a subset of the National Health Insurance Database, which is released by the National Health Research Institutes in Taiwan. The 2010 LHIRD, in which 1 million beneficiaries are randomly sampled from National Health Insurance (NHI) system, comprises the claims data of outpatient, admission, and prescription from 2009 to 2013. The NHI system is a single-payer social insurance system, and the coverage was approximately 98% in 2010. To protect the privacy of patients and care providers, the personnel identification numbers are scrambled for de-identification. The study was approved by the Ethical Review Board of Chung Shan Medical University Hospital (CSMU No.: 18096).

### 2.2. Patient Selection (Exposure of UTI Infection)

This was a retrospective cohort study. We identified patients (*n* = 140,308) who visited a hospital or were hospitalized for UTI (ICD-9: 599.0, 595.0, 595.9, and 590) and had antibiotic therapy (cephalosporins, quinolone antibacterial, sulfonamides and trimethoprim, ampicillin, amoxicillin, and nitrofurantoin) in the same visit during 2009–2013 as the exposure group. To ensure that only newly identified UTI was included, prevalent cases of UTI in 2009 were excluded. Furthermore, patients newly diagnosed with UTI in 2013 were excluded because they were only followed up for less than 1 year. The index date was the first date of UTI visit or admission; additional exclusion criteria included patients aged less than 20 years old, those having any cancer, those who died, and those who underwent urine examination within 6 months after the index date. A total of 38,084 patients were diagnosed with UTI in this study.

### 2.3. Propensity Score Matching (PSM)

A potential confounding bias exists in the observational study design. Propensity score matching (PSM) was used to diminish this bias. We used a logistic regression model to estimate the probability (propensity score) of UTI, using such predictors as age, the Charlson Comorbidity Index (CCI), and other comorbidities (hypertension (ICD-9: 401–405), diabetes (ICD-9: 250), dyslipidemia (ICD-9: 272), rheumatic diseases (ICD-9: 714, 710, 720, 696.0, and 696.1), coronary artery disease (ICD-9: 410–414), chronic obstructive pulmonary disease (COPD, ICD-9: 490–492, 493–496), and chronic kidney disease (CKD, ICD-9: 585)). The control (non-UTI exposure during 2009–2013) and study groups were 1:2 propensity-score-matched on the basis of sex because of the specific cancer sites in different genders. The greedy algorithm of PSM was applied using SAS macro [11].

### 2.4. Outcome Measurement of Cancer Event

Subsequent cancer events were identified according to ICD-9: 140–208 for ≥2 outpatient visits or ≥1 admission. Major cancer sites reported in Taiwan were considered for subevent analysis, including colorectal (ICD-9: 153–154), liver (ICD-9: 155), lung (ICD-9: 162), and breast (ICD-9: 174), bladder (ICD-9: 188), kidney (ICD-9: 189), male genital organs (ICD-9: 185–187), female genital organs (ICD-9: 179–184), and prostate (ICD-9: 185) cancers. All individuals were followed up from the index date until diagnosed with any cancer, death, or end of study (31 December 2013).

### 2.5. Statistical Analysis

All analyses were performed after stratifying the data by sex because gender-specific cancer sites were analyzed in this study. Chi-squared test was used to analyze the homogeneity of category variables, and univariate and multivariate Cox regression models were conducted to estimate the crude and adjusted hazard ratio (aHR) (95% confidence interval (CI)). All statistical analyses were performed using SAS (version 9.4; SAS Institute, Cary, NC, USA). *p* less than 0.05 indicated statistical significance.

## 3. Results

**Figure 1:**
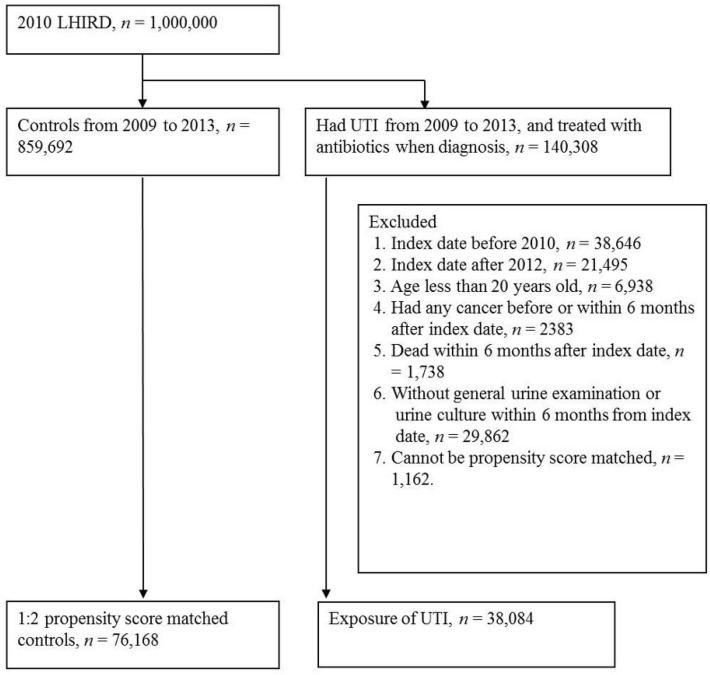
Flow chart for patient selection.

We identified 38,084 patients diagnosed with UTI from 2010 to 2012 and a total of 76,168 propensity score-matched controls to explore their sequential cancer risk after the index date (Figure 1). The baseline characteristics among the UTI and non-UTI groups stratified by sex are listed in Table 1. After PSM, no significant difference was observed in the distributions of age group, CCI group, and comorbidities (hypertension, diabetes hyperlipidemia, rheumatic diseases, coronary artery disease, chronic obstructive pulmonary disease (COPD), and chronic kidney disease (CKD) in both genders. More female UTI cases (the sex ratio, F:M = 31,172:6,912) were observed, especially in women aged 20–44 and 45–65 years. The median of follow-up time was 25 months (Max: 42), because the index date (start point of follow-up) was 6 months after UTI.

The age–sex stratified incidence rate (per 10,000 person-months) and adjusted hazard risks of specific-site cancer (colorectal, liver, lung, genital organs, bladder, kidney, male prostate, female breast cancer, and any cancer) in patients with UTI are presented in Table 2. For men aged 20–64 years, significant aHRs were observed in any cancers (aHR = 1.37, 95% CI = 1.02–1.86), bladder cancer (aHR = 12.10, 95% CI = 2.70–54.19), and kidney cancer (aHR = 5.20, 95% CI = 1.01–26.82). For men aged ≥65 years, the associations were observed in any cancer (aHR = 1.29, 95% CI = 1.08–1.54), colorectal cancer (aHR = 1.59, 95% CI = 1.01–2.52), genital organ cancer (aHR = 2.37, 95% CI = 1.55–3.64), bladder cancer (aHR = 28.60, 95% CI = 6.80–120.28), kidney cancer (aHR = 3.85, 95% CI = 1.42–10.42), and prostate cancer (aHR = 2.44, 95% CI = 1.59–3.74). For women aged 20–64 years, the significant aHRs were estimated in liver cancer (aHR = 2.44, 95% CI = 1.59–3.74), bladder cancer (aHR = 30.02, 95% CI = 3.97–227.28), and kidney cancer (aHR = 2.90, 95% CI = 1.24–6.78). For women aged ≥65 years, the significantly increased aHRs were observed in any cancer (aHR = 1.30, 95% CI = 1.11–1.53), liver cancer (aHR = 1.54, 95% CI = 1.02–2.33), bladder cancer (aHR = 2.33, 95% CI = 1.01–5.42), and kidney cancer (aHR = 3.40, 95% CI = 1.34–8.64).

The significantly increased HRs of any cancer for UTI exposure in men (crude HR = 1.27, 95% CI = 1.09–1.48; aHR = 1.32, 95% CI = 1.12–1.54) and women (crude HR = 1.21, 95% CI = 1.09–1.36; aHR = 1.21, 95% CI = 1.08–1.35) are indicated in Table 3 and Table 4. Additionally, we also demonstrated that in individuals aged ≥65 years, a CCI score of ≥5 indicated higher risk of cancer incidence.

In Table 5, no interaction effect between UTI and pneumonia on cancer incidence was observed. We demonstrated the dose response of antibiotic prescriptions on cancer incidence in men; the significant p for trend was observed in any cancer (*p* = 0.0130) and bladder cancer (*p* = 0.0066). However, no significant p trends were observed in women (Table 6).

**Figure A1:**
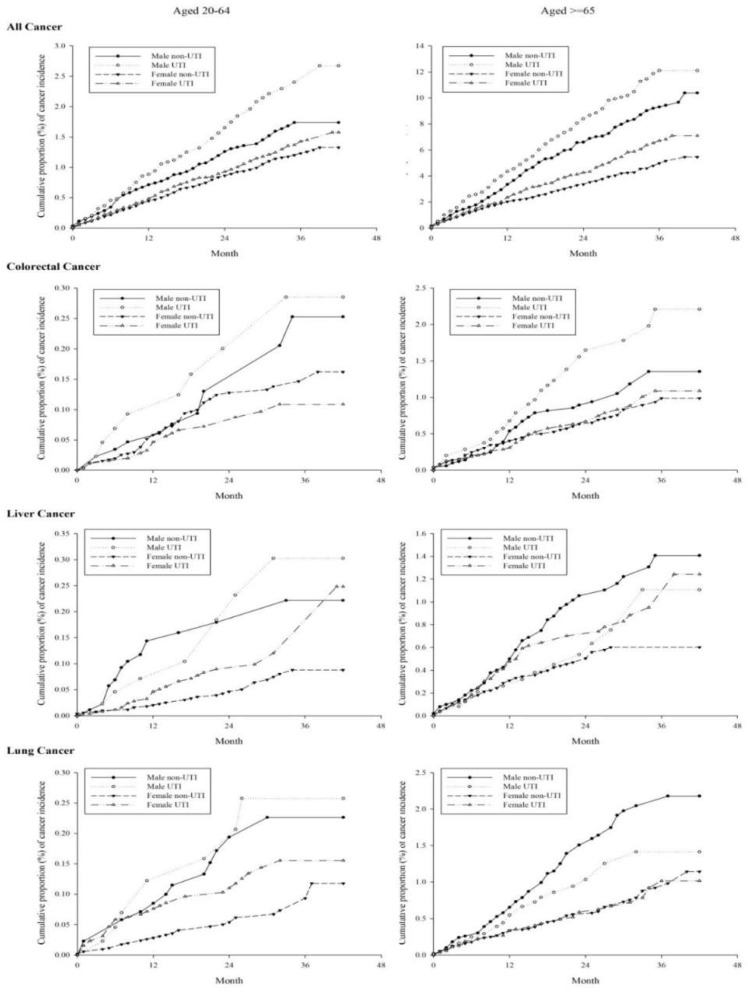

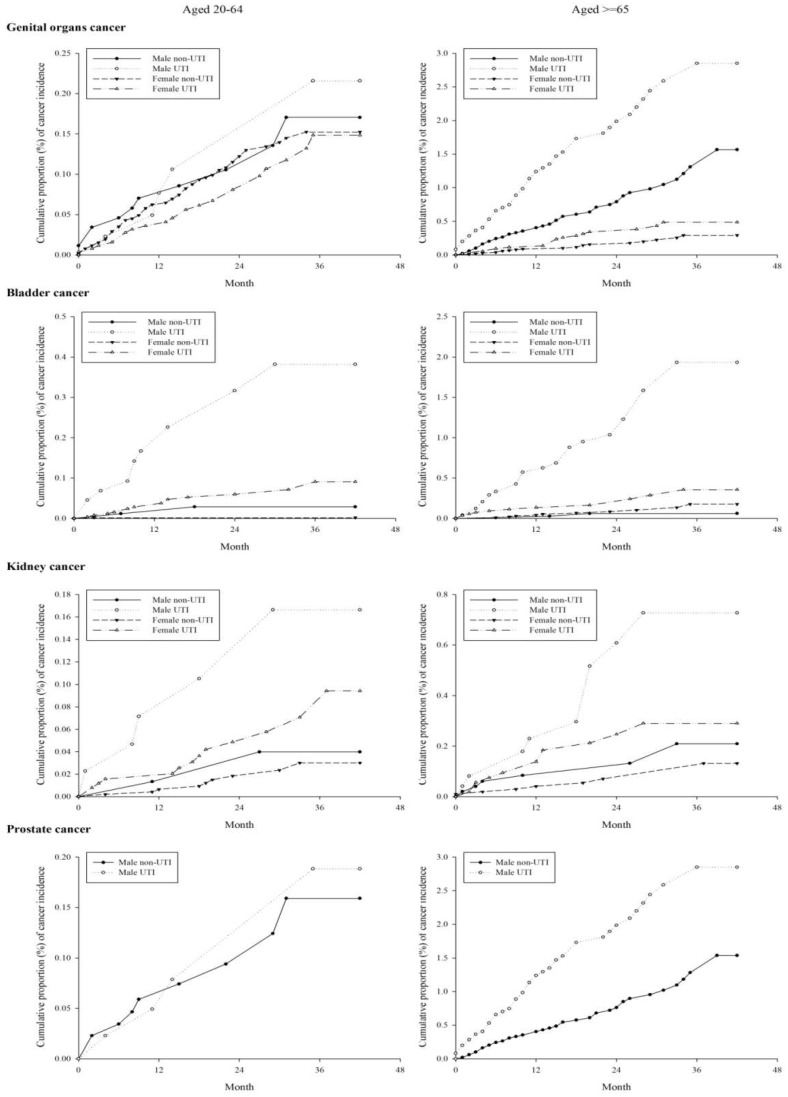

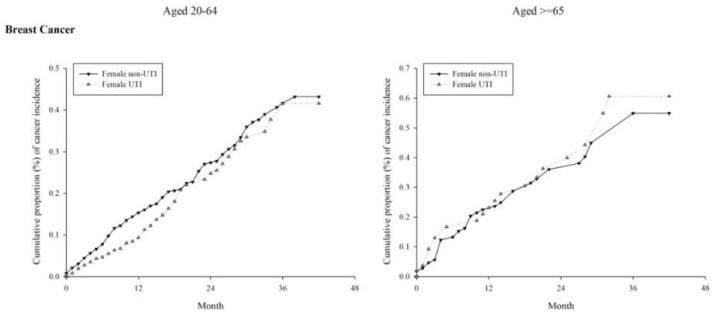
Kaplan-Meier curve for specific cancer risk in patients with/without UTI infection stratified by age group.

The Kaplan–Meier curve for specific cancer risk among the study groups are shown in Appendix A Figure A1. Any cancer risk was under proportional hazard assumption; however, bladder and kidney cancer risks were modified after 24 and 18 months, respectively, in the elder (≥65 years old) population.

## 4. Discussion

Per the analysis results presented in Figure A1, risk significantly increased not only in GUC but also in any-cancer development. Males older than 65 years exhibited a negative association between UTI and lung cancer risk (Figure A1). In previous reports, cephalosporins and quinolones were associated with antitumor properties [12,13]. The incidence of lung cancer in middle-aged men with UTI may be reduced through antibiotic treatments, and this hypothesis requires more evidence before it can be confirmed.

UTI and pneumonia are clinically common complications, and the results of this work also showed a high correlation between UTI and pneumonia (*p* < 0.001) (Table 3). Some reports have revealed that pneumonia has a high correlation with lung cancer, and Marcus et al. provided evidence of increased lung cancer risk among history of pneumonia rather than immunodeficiency [14]. The association between pneumonia and any cancer is rarely reported. The incidence of association between pneumonia and any cancer was low in both men and women in this study (*p* > 0.05).

UTI is particularly associated with the bladder and kidney cancer in both men and women (Table 2). The significant and large relative risk was found whether in young (20–64 y/o) or elderly (≥65 y/o) population. This evidence reinforces the study hypothesis that UTI directly increase the risk in bladder and kidney cancers, which is consistent with Anderson-Otunu’s 2016 report [6]. Vermeulen (2015) revealed that UTI are associated with a high risk of urinary bladder cancer in postmenopausal women, especially in women who smoke or had smoked [7]. In males, the obstructive urinary symptoms induced by benign prostatic hyperplasia include difficulty in urination and urine retention, resulting in UTI caused from urinary stasis [15].

Similarly, Table 6 indicates that the use of antibiotics in the course of UTI treatments increases the risk of bladder and kidney cancers. We analyzed the tumor incidence risk between UTI and pneumonia after antibiotic treatment (Table 5). The correlation coefficient between antibiotic treatment and tumor incidence was considerably low. However, a significant increase was observed in the incidence of bladder cancer in men after antibiotic treatment for more than 7 days (Table 6). These results are contrary to many research conclusions, and most antibiotics such as cephalosporins, quinolones, and ampicillins are considered as a kind of cancer treatment drug rather than a carcinogen [12,13,16]. However, no report has clarified whether beta-lactams, amoxicillin, sulfamethoxazole, and trimethoprim are carcinogenic or have antitumor properties. Only nitrofurantoin is a possible carcinogen due to its genotoxic and carcinogenic potential structures, despite its antimicrobial property [17,18,19,20]. In Kimura’s report (2016), nitrofurantoin does not exert sufficient renal carcinogen responses even after 28 days of administration [21]. In a recent study on the structure-related genotoxicity of nitrofurantoin, a new evidence revealed that nitrofurantoin does not increase the mutation frequency in the experimental mice. Nevertheless, nitrofurantoin presents no genotoxicity without oxidative stress [22]. This report provided a safe basis for nitrofuran compound development. The gold standard for UTI diagnosis is isolation and quantification of pathogens in the presence of symptoms and obtaining the antibiotic sensitivity pattern to allow specific treatments. However, in this observational study, we cannot provide the evidence of carcinogen from antibiotics. The length (days) of antibiotics usage also correlated with the severity of infection, that might be the risk factor of cancer. However, the appropriate drug dosage should be based on the severity, characteristics and treatment of the infection situation. Therefore, we should consider antibiotic doses in the treatment of UTI under the premise of controlling infection and avoiding drug resistance. Thus, further research needs to clarify this issue in the future. Women after UTI have higher liver cancer risk as reported in this work, especially approximately 30 months after infection. UTI infection is positively associated with GUC, which is consistent with previous findings. Therefore, this study posits a nonpathogenic causal relationship with UTI symptoms.

Our research has some limitations. First, the database used does not contain information on patients’ clinical presentation, for example, the severity of UTI, personal behavioral information such as drinking, smoking habits, and body mass index, and microbiological culture data that might affect UTI occurrence. Second, the NHI system is limited to the population of Taiwan. Our findings reflect the situation in Taiwan, but it may not be applicable to Western populations. These confounding factors might have influenced the results.

## 5. Conclusions

In conclusion, UTI are highly correlated with the incidence of all tumors. Clinicians should therefore refer to tumors screening in UTI patients, especially older patients, and not just patients with GUC. In UTI treatment, especially when multiple pathogenic factors are entailed, antibiotics must be used cautiously, and the time and dose of antibiotics should be minimized. Because UTI may increase the incidence of genital organ, bladder, kidney, male colorectal, prostate, and female liver cancer, knowledge about preventing UTI such as proper drinking water, exercise, and toilet habits should be enhanced in educating the general public.

## Figures and Tables

**Table 1:** Baseline characteristics of the study population.

	Male		*p* Value	Female		*p* Value
	Non-UTI N = 13,824	UTI N = 6,912		Non-UTI N = 62,344	UTI N = 31,172	
Age			0.8332			0.4579
20–44	4355 (31.5%)	2201 (31.84%)		31112 (49.90%)	15513 (49.77%)	
45–65	4411 (31.91%)	2209 (31.96%)		20540 (32.95%)	10212 (32.76%)	
≥65	5058 (36.59%)	2502 (36.20%)		10692 (17.15%)	5447 (17.47%)	
CCIs			0.8214			0.1808
0	6503 (47.04%)	3260 (47.16%)		37784 (60.61%)	18906 (60.65%)	
1–2	5215 (37.72%)	2604 (37.67%)		20116 (32.27%)	9961 (31.95%)	
3–4	1670 (12.08%)	846 (12.24%)		3765 (6.04%)	1921 (6.16%)	
≥5	436 (3.15%)	202 (2.92%)		679 (1.09%)	384 (1.23%)	
Co-morbidity						
Hypertension	5270 (38.12%)	2661 (38.50%)	0.5993	13847 (22.21%)	6963 (22.34%)	0.6605
Diabetes	2484 (17.97%)	1248 (18.06%)	0.8781	7391 (11.86%)	3730 (11.97%)	0.6221
Hyperlipidemia	2842 (20.56%)	1414 (20.46%)	0.8648	10363 (16.62%)	5189 (16.65%)	0.9258
Rheumatic diseases	399 (2.89%)	207 (2.99%)	0.6619	2650 (4.25%)	1317 (4.22%)	0.8544
Coronary artery disease	1943 (14.06%)	957 (13.85%)	0.6814	4702 (7.54%)	2367 (7.59%)	0.7795
COPD	2083 (15.07%)	1047 (15.15%)	0.8801	4970 (7.97%)	2594 (8.32%)	0.0645
CKD	470 (3.40%)	231 (3.34%)	0.8279	723 (1.16%)	397 (1.27%)	0.1312
Pneumonia	254 (1.84%)	628 (9.09%)	<0.0001	388 (0.62%)	716 (2.30%)	<0.0001

UTI: urinary tract infection; CCI: Charlson Comorbidity Index.

**Table 2:** Age stratified cancer incidence rate (per 10,000 person months) and adjusted hazard ratio (95% C.I.) ^†^ in patients with UTI exposure by cancer site.

	20–64 y/o		aHR	≥65 y/o		aHR
	Incidence Rate			Incidence Rate		
	Non-UTI	UTI		Non-UTI	UTI	
**Male**						
All cancer	5.05	6.93	**1.37 (1.02–1.86)**	27.70	35.40	**1.29 (1.08–1.54)**
Colorectal	0.58	0.77	1.32 (0.54–3.23)	3.78	5.96	**1.59 (1.01–2.52)**
Liver	0.72	0.77	1.05 (0.44–2.47)	4.04	2.61	0.65 (0.36–1.19)
Lung	0.67	0.77	1.46 (0.75–2.86)	5.98	4.10	1.13 (0.75–1.70)
Genital organs	0.48	0.48	1.05 (0.36–3.09)	3.52	8.39	**2.37 (1.55–3.64)**
Bladder	0.10	1.15	**12.10 (2.70–54.19)**	0.18	5.03	**28.60 (6.80–120.28)**
Kidney	0.10	0.48	**5.20 (1.01–26.82)**	0.53	2.05	**3.85 (1.42–10.42)**
Prostate	0.43	0.38	0.96 (0.29–3.15)	3.43	8.39	**2.44 (1.59–3.74)**
**Female**						
All cancer	3.52	3.95	1.13 (0.97–1.32)	14.49	18.96	**1.30 (1.11–1.53)**
Colorectal	0.45	0.32	0.70 (0.42–1.17)	2.76	2.91	1.06 (0.71–1.57)
Liver	0.21	0.36	**1.75 (1.00–3.04)**	1.94	3.06	**1.54 (1.02–2.33)**
Lung	0.25	0.47	1.22 (0.86–1.71)	2.56	2.52	0.75 (0.43–1.32)
Genital organs	0.46	0.36	0.79 (0.49–1.28)	0.74	1.38	1.86 (0.98–3.56)
Bladder	0.01	0.24	**30.02 (3.97–227.28)**	0.39	0.92	**2.34 (1.01–5.42)**
Kidney	0.07	0.21	**2.90 (1.24–6.78)**	0.27	0.92	**3.40 (1.34–8.64)**
Breast	1.16	1.04	0.90 (0.68–1.21)	1.51	1.68	1.10 (0.65–1.85)

^†^ adjusted for age (per 1 year), CCI score, and co-morbidities (including hypertension, diabetes, hyperlipidemia, rheumatic disease, coronary artery disease, COPD, CKD, and pneumonia. Bold font indicates statistical significance (*p* < 0.05).

**Table 3:** Adjusted hazard ratio of all cancer in males.

	Crude HR	95% C.I.	*p* Value	aHR ^†^	95% C.I.	*p* Value
UTI (Ref: No)						
Yes	1.27	1.09–1.48	0.0023	1.32	1.12–1.54	0.0007
Age (Ref: 20-44)						
45-65	5.21	3.50–7.76	<0.0001	4.69	3.14–7.03	<0.0001
≥65	16.37	11.26–23.79	<0.0001	12.32	8.29–18.31	<0.0001
CCIs score (Ref: 0)						
1–2	2.34	1.94–2.82	<0.0001	1.28	1.03–1.58	0.0230
3–4	3.87	3.11–4.81	<0.0001	1.50	1.13–1.99	0.0046
≥5	5.42	3.92–7.49	<0.0001	1.88	1.24–2.84	0.0027
Co-morbidity						
Hypertension	2.69	2.31–3.14	<0.0001	1.12	0.94–1.34	0.1901
Diabetes	1.76	1.49–2.09	<0.0001	0.90	0.73–1.10	0.2867
Hyperlipidemia	1.38	1.16–1.64	0.0002	0.93	0.77–1.12	0.4413
Rheumatic diseases	1.31	0.89–1.94	0.1747	1.00	0.67–1.48	0.9981
Coronary artery disease	2.44	2.06–2.89	<0.0001	1.25	1.04–1.49	0.0165
COPD	2.02	1.70–2.40	<0.0001	1.05	0.86–1.27	0.6569
CKD	2.21	1.62–3.03	<0.0001	0.98	0.70–1.37	0.8845
Pneumonia	2.15	1.61–2.88	<0.0001	0.95	0.70–1.29	0.7612

^†^ adjusted for UTI infection, age group, CCI score, and co-morbidities (including hypertension, diabetes, hyperlipidemia, rheumatic disease, coronary artery disease, COPD, CKD, and pneumonia.

**Table 4:** Adjusted hazard ratio of all cancer in Female.

	Crude HR	95% C.I.	*p* Value	aHR ^†^	95% C.I.	*p* Value
UTI (Ref: No)						
Yes	1.21	1.09–1.36	0.0007	1.21	1.08–1.35	0.0009
Age (Ref: 20-44)						
45–65	4.68	3.94–5.57	<0.0001	4.41	3.68–5.27	<0.0001
≥65	10.78	9.09–12.78	<0.0001	9.00	7.37–11.00	<0.0001
CCIs score (Ref: 0)						
1–2	2.16	1.92–2.44	<0.0001	1.24	1.08–1.43	0.0023
3–4	3.86	3.27–4.57	<0.0001	1.40	1.13–1.74	0.0024
≥5	5.47	4.02–7.44	<0.0001	1.54	1.03–2.29	0.0349
Co-morbidity						
Hypertension	3.08	2.76–3.43	<0.0001	1.11	0.97–1.26	0.1444
Diabetes	2.39	2.10–2.71	<0.0001	0.95	0.81–1.11	0.4982
Hyperlipidemia	1.94	1.72–2.19	<0.0001	0.88	0.78–1.01	0.0774
Rheumatic diseases	1.28	1.00–1.63	0.0478	0.93	0.72–1.18	0.5332
Coronary artery disease	2.51	2.18–2.91	<0.0001	1.05	0.90–1.23	0.5270
COPD	1.63	1.39–1.93	<0.0001	0.91	0.76–1.09	0.3035
CKD	3.76	2.84–4.97	<0.0001	1.51	1.09–2.10	0.0141
Pneumonia	2.18	1.52–3.13	<0.0001	0.97	0.67–1.41	0.8892

^†^ adjusted for UTI infection, age group, CCI score, and co-morbidities (including hypertension, diabetes, hyperlipidemia, rheumatic disease, coronary artery disease, COPD, CKD, and pneumonia.

**Table 5:** Adjusted hazard risk ^†^ of cancer in patients with UTI or Pneumonia.

	Non-UTI and Non-Pneumonia	Only UTI	Only Pneumonia	UTI Combined Pneumonia	*p* for Interaction
**Male**					
All cancer	Reference	1.35 (1.15–1.59)	1.24 (0.75–2.06)	1.14 (0.80–1.64)	0.2270
Colorectal	Reference	1.54 (0.99–2.38)	1.90 (0.58–6.19)	1.76 (0.75–4.16)	0.4963
Liver	Reference	0.86 (0.52–1.41)	1.12 (0.27–4.65)	0.23 (0.03–1.69)	0.2585
Lung	Reference	0.79 (0.50–1.24)	1.32 (0.48–3.68)	0.78 (0.31–1.93)	0.6723
Genital organs	Reference	2.23 (1.49–3.35)	0.60 (0.08–4.36)	1.25 (0.49–3.17)	0.9541
Bladder	Reference	21.64 (7.71–60.77)	Can not estimate	7.63 (1.37–42.42)	0.9879
Kidney	Reference	4.10 (1.72–9.80)	Can not estimate	3.84 (0.78–18.87)	0.9908
Prostate	Reference	2.29 (1.51–3.45)	0.61 (0.08–4.45)	1.28 (0.51–3.26)	0.9426
**Female**					
All cancer	Reference	1.20 (1.08–1.35)	0.85 (0.42–1.72)	1.24 (0.81–1.90)	0.6554
Colorectal	Reference	0.92 (0.68–1.26)	0.68 (0.10–4.93)	0.35 (0.05–2.50)	0.6741
Liver	Reference	1.57 (1.12–2.21)	0.72 (0.10–5.19)	1.76 (0.70–4.41)	0.6862
Lung	Reference	1.26 (0.91–1.75)	2.13 (0.676.79)	1.95 (0.78–4.85)	0.6684
Genital organs	Reference	1.03 (0.70–1.52)	Can not estimate	1.63 (0.39–6.77)	0.9735
Bladder	Reference	4.84 (2.39–9.80)	Can not estimate	4.60 (0.58–36.22)	0.9944
Kidney	Reference	3.43 (1.81–6.51)	6.44 (0.83–50.19)	Can not estimate	0.9768
Breast	Reference	0.96 (0.75–1.24)	0.79 (0.11–5.66)	0.43 (0.06–3.08)	0.6890

^†^ adjusted for age group, CCI score, and co-morbidities (including hypertension, diabetes, hyperlipidemia, rheumatic disease, coronary artery disease, COPD, CKD, and pneumonia

**Table 6:** Adjusted hazard risk ^†^ of cancer for dosage (days) trend of antibiotics prescriptions in patients with UTI.

	Days of Antibiotics Prescriptions within 6 Months before Index Date			*p* for Trend
	1–6 days	7–13 days	≥14 days	
**Male**	*n* = 17241	*n* = 1940	*n* = 1555	
All cancer	Reference	1.02 (0.75–1.40)	1.45 (1.09–1.91)	0.0130
Colorectal	Reference	1.16 (0.54–2.46)	1.05 (0.49–2.23)	0.8669
Liver	Reference	0.93 (0.32–2.72)	1.35 (0.51–3.56)	0.5874
Lung	Reference	1.43 (0.60–3.41)	1.39 (0.58–3.30)	0.4213
Genital organs	Reference	0.81 (0.37–1.77)	1.44 (0.78–2.66)	0.2860
Bladder	Reference	1.23 (0.50–3.02)	2.73 (1.32–5.64)	0.0066
Kidney	Reference	0.68 (0.18–2.55)	1.14 (0.37–3.54)	0.8995
Prostate	Reference	0.82 (0.38–1.78)	1.36 (0.73–2.54)	0.3822
**Female**	*n* = 80515	*n* = 9014	*n* = 3987	
All cancer	Reference	1.08 (0.88–1.32)	1.10 (0.87–1.40)	0.3605
Colorectal	Reference	1.00 (0.54–1.85)	1.31 (0.68–2.54)	0.4775
Liver	Reference	1.19 (0.67–2.10)	1.12 (0.58–2.19)	0.6352
Lung	Reference	1.53 (0.87–2.69)	1.49 (0.78–2.85)	0.1511
Genital organs	Reference	2.06 (1.10–3.85)	0.33 (0.08–1.41)	0.7254
Bladder	Reference	0.41 (0.12–1.44)	2.30 (1.01–5.25)	0.1283
Kidney	Reference	2.34 (0.97–5.66)	1.73 (0.58–5.20)	0.1741
Breast	Reference	1.13 (0.70–1.83)	1.42 (0.81–2.49)	0.2356

^†^ adjusted for age group, CCI score, and co-morbidities (including hypertension, diabetes, hyperlipidemia, rheumatic disease, coronary artery disease, COPD, CKD, and pneumonia.

## References

[1] Nicolle L.E. (2013). Urinary tract infection. Crit. Care Clin..

[2] Sun L.M., Lin C.L., Liang J.A., Liu S.H., Sung F.C., Chang Y.J., Kao C.H. (2013). Urinary tract infection increases subsequent urinary tract cancer risk: A population-based cohort study. Cancer Sci..

[3] Erdem I., Kara Ali R., Ardic E., Elbasan Omar S., Mutlu R., Topkaya A.E. (2018). Community-acquired lower urinary tract infections: Etiology, antimicrobial resistance, and treatment results in female patients. J. Glob. Infect. Dis..

[4] Arnold J.J., Hehn L.E., Klein D.A. (2016). Common questions about recurrent urinary tract infections in women. Am. Family Phys..

[5] Rowe T.A., Juthani-Mehta M. (2014). Diagnosis and management of urinary tract infection in older adults. Infect. Disease Clin. N. Am..

[6] Anderson-Otunu O., Akhtar S. (2016). Chronic infections of the urinary tract and bladder cancer risk: A systematic review. Asian Pac. J. Cancer Prev. APJCP.

[7] Vermeulen S.H., Hanum N., Grotenhuis A.J., Castano-Vinyals G., van der Heijden A.G., Aben K.K., Mysorekar I.U., Kiemeney L.A. (2015). Recurrent urinary tract infection and risk of bladder cancer in the nijmegen bladder cancer study. Br. J. Cancer.

[8] Dune T.J., Price T.K., Hilt E.E., Thomas-White K.J., Kliethermes S., Brincat C., Brubaker L., Schreckenberger P., Wolfe A.J., Mueller E.R. (2017). Urinary symptoms and their associations with urinary tract infections in urogynecologic patients. Obstet. Gynecol..

[9] Russell B., Garmo H., Beckmann K., Stattin P., Adolfsson J., Van Hemelrijck M. (2018). A case-control study of lower urinary-tract infections, associated antibiotics and the risk of developing prostate cancer using pcbase 3.0. PLoS ONE.

[10] Chastain D.B., King S.T., Stover K.R. (2018). Rethinking urinary antibiotic breakpoints: Analysis of urinary antibiotic concentrations to treat multidrug resistant organisms. BMC Res. Notes.

[11] Parsons L.S. Performing a 1: N case-control match on propensity score. Proceedings of the 29th Annual SAS Users Group International Conference.

[12] Maj M., Bajek A., Nalejska E., Porowinska D., Kloskowski T., Gackowska L., Drewa T. (2017). Influence of mesenchymal stem cells conditioned media on proliferation of urinary tract cancer cell lines and their sensitivity to ciprofloxacin. J. Cell. Biochem..

[13] Batalha P.N., Vieira de Souza M.C., Pena-Cabrera E., Cruz D.C., da Costa Santos Boechat F. (2016). Quinolones in the search for new anticancer agents. Curr. Pharm. Des..

[14] Marcus J.L., Leyden W.A., Chao C.R., Horberg M.A., Klein D.B., Quesenberry C.P., Towner W.J., Silverberg M.J. (2017). Immunodeficiency, aids-related pneumonia, and risk of lung cancer among hiv-infected individuals. AIDS.

[15] Michaud D.S. (2007). Chronic inflammation and bladder cancer. Urol. Oncol..

[16] Ferraz R., Costa-Rodrigues J., Fernandes M.H., Santos M.M., Marrucho I.M., Rebelo L.P., Prudencio C., Noronha J.P., Petrovski Z., Branco L.C. (2015). Antitumor activity of ionic liquids based on ampicillin. ChemMedChem.

[17] Hasegawa R., Murasaki G., St John M.K., Zenser T.V., Cohen S.M. (1990). Evaluation of nitrofurantoin on the two stages of urinary bladder carcinogenesis in the rat. Toxicology.

[18] Kijima A., Ishii Y., Takasu S., Matsushita K., Kuroda K., Hibi D., Suzuki Y., Nohmi T., Umemura T. (2015). Chemical structure-related mechanisms underlying in vivo genotoxicity induced by nitrofurantoin and its constituent moieties in gpt delta rats. Toxicology.

[19] Williams G.M. (1980). Classification of genotoxic and epigenetic hepatocarcinogens using liver culture assays. Ann. N. Y. Acad. Sci..

[20] International Agency for Research on Cancer (1994). Monographs on the Evaluation of Carcinogen Risk to Humans: Some Industrial Chemicals.

[21] Kimura M., Mizukami S., Watanabe Y., Hasegawa-Baba Y., Onda N., Yoshida T., Shibutani M. (2016). Disruption of spindle checkpoint function in rats following 28 days of repeated administration of renal carcinogens. J. Toxicol. Sci..

[22] Tsuchiya T., Kijima A., Ishii Y., Takasu S., Yokoo Y., Nishikawa A., Yanai T., Umemura T. (2018). Role of oxidative stress in the chemical structure-related genotoxicity of nitrofurantoin in nrf2-deficient gpt delta mice. J. Toxicol. Pathol..

